# HLA typing of patients who developed subacute thyroiditis and Graves’ disease after SARS-CoV-2 vaccination: a case report

**DOI:** 10.1186/s12902-023-01287-5

**Published:** 2023-03-07

**Authors:** Shigemitsu Yasuda, Seiya Suzuki, Shinnosuke Yanagisawa, Hideo Morita, Akifumi Haisa, Atsushi Satomura, Ritsuko Nakajima, Yoichi Oikawa, Ikuo Inoue, Akira Shimada

**Affiliations:** Department of Endocrinology and Diabetes, Saitama Medical University, Morohongo 38, Moroyama, Iruma-gun, Saitama 350-0495 Japan

**Keywords:** Subacute thyroiditis, COVID-19, SARS-CoV-2 vaccination, HLA, Graves’ disease, Case report

## Abstract

**Background:**

Cases of subacute thyroiditis (SAT) after severe acute respiratory syndrome coronavirus 2 (SARS-CoV-2) vaccination have been reported. A human leukocyte antigen (HLA) allele, HLA-B*35, appears to be involved in the pathogenesis of SAT.

**Case presentation:**

We conducted HLA typing of one patient with SAT and another with both SAT and Graves’ disease (GD), which developed after SARS-CoV-2 vaccination. Patient 1, a 58-year-old Japanese man, was inoculated with a SARS-CoV-2 vaccine (BNT162b2; Pfizer, New York, NY, USA). He developed fever (38 °C), cervical pain, palpitations, and fatigue on day 10 after vaccination. Blood chemistry tests revealed thyrotoxicosis and elevated serum C-reactive protein (CRP) and slightly increased serum antithyroid-stimulating antibody (TSAb) levels. Thyroid ultrasonography revealed the characteristic findings of SAT. Patient 2, a 36-year-old Japanese woman, was inoculated twice with a SARS-CoV-2 vaccine (mRNA-1273; Moderna, Cambridge, MA, USA). She developed fever (37.8 °C) and thyroid gland pain on day 3 after the second vaccination. Blood chemistry tests revealed thyrotoxicosis and elevated serum CRP, TSAb, and antithyroid-stimulating hormone receptor antibody levels. Fever and thyroid gland pain persisted. Thyroid ultrasonography revealed the characteristic findings of SAT (i.e., slight swelling and a focal hypoechoic area with decreased blood flow). Prednisolone treatment was effective for SAT. However, thyrotoxicosis causing palpitations relapsed thereafter, for which thyroid scintigraphy with ^99m^technetium pertechnetate was conducted, and the patient was diagnosed with GD. Thiamazole treatment was then initiated, which led to improvement in symptoms.

**Conclusion:**

HLA typing revealed that both patients had the HLA-B*35:01, -C*04:01, and -DPB1*05:01 alleles. Only patient 2 had the HLA-DRB1*11:01 and HLA-DQB1*03:01 alleles. The HLA-B*35:01 and HLA-C*04:01 alleles appeared to be involved in the pathogenesis of SAT after SARS-CoV-2 vaccination, and the HLA-DRB1*11:01 and HLA-DQB1*03:01 alleles were speculated to be involved in the postvaccination pathogenesis of GD.

## Background

Subacute thyroiditis (SAT) is an inflammatory condition of the thyroid gland with characteristic presentation that generally occurs after an upper respiratory tract infection. SAT is thought to be caused by viral infection of the thyroid gland. Many viruses such as coxsackievirus, adenovirus, paramyxovirus, morbillivirus, and severe acute respiratory syndrome coronavirus 2 (SARS-CoV-2) are involved in the onset of SAT [1, 2]. Susceptibility to SAT has been associated with certain human leukocyte antigen (HLA) alleles including HLA-B*35:01, HLA-B*18:01, and HLA-C*04:01 [3–5]. Furthermore, the HLA-DPB1*05:01 and *HLA-A2* alleles are known to be involved in the susceptibility to Graves’ disease (GD), an autoimmune disorder affecting the thyroid gland, in the Japanese population [6]. Recently, some cases of SAT [7] and GD after the emergence of coronavirus disease 2019 (COVID-19) have been reported in several countries. Regarding COVID-19-induced SAT, atypical SAT that frequently follows a painless course was rarely reported [8]. Moreover, the involvement of the HLA-B*35 allele was suggested in the pathogenesis of SAT after COVID-19 [9]. Not only COVID-19 but also the SARS-CoV-2 vaccine was also reported to cause SAT [10]. The co-presence of the HLA-B*35 and HLA-C*04 alleles can play an important role in the post-SARS-CoV-2 vaccination pathogenesis of SAT [11, 12]. To the best of our knowledge, however, a detailed investigation of these issues has not yet been conducted. The development of SAT after other vaccinations, such as the influenza vaccine, have also been reported [13, 14].

The systematic review of Ippolito et al. [10] reported on 51 patients who developed SAT after SARS-CoV-2 vaccination in some countries in Europe, Asia, and North America, with predominance of females (74.5%), median age of 39.5 years, and symptoms very similar to those found in typical SAT. In addition, Jafarzadeh et al. [15] investigated the classes of the SARS-CoV-2 vaccines inducing SAT (i.e., mRNA, inactivated, and vector-based) and found that 1) the mRNA SARS-CoV-2 vaccine was mostly responsible for SAT, as with GD after SARS-CoV-2 vaccination, 2) times from vaccination to the onset of SAT and GD were about 10 days and about 10–14 days, respectively, and 3) both SAT and GD developed after the first and second vaccinations.

We encountered two patients at our institution who developed SAT after SARS-CoV-2 vaccination. One patient, who was positive for antithyroid-stimulating antibody (TSAb), developed SAT but not GD. Another patient who was positive for antithyroid-stimulating hormone receptor antibody (TRAb), developed both SAT and GD. To date, a limited number of cases of SAT and GD after SARS-CoV-2 vaccination have been reported [16, 17]. Herein, we report on two aforementioned patients for whom we also conducted HLA typing.

## Case presentation

### Patient 1

A 58-year-old Japanese man was diagnosed with hypertension at another clinic at the age of 40 years. He began taking amlodipine, a calcium channel blocker, orally at a dose of 2.5 mg/day, which continued thereafter. Treatment and family history indicated the absence of thyroid disorders. The patient had never experienced adverse reactions from vaccination or adverse events from any drug. He had no smoking history. On the next day after SARS-CoV-2 vaccination (BNT162b2, Pfizer, New York, NY, USA), he developed fever (38.0 °C) that resolved spontaneously. On day 10 after vaccination, fever (38.0 °C) recurred along with cervical pain and fatigue. On day 16, the patient sought medical attention at Saitama Medical University Hospital because of unimproved symptoms. The polymerase chain reaction (PCR) test for COVID-19 was negative. Routine blood chemistry tests indicated a normal white blood cell (WBC) count of 5240 cells/μL (reference range: 3300–8600 cells/μL) and a markedly increased serum C-reactive protein (CRP) level of 7.19 mg/dL (reference range: < 0.14 mg/dL).

Regarding thyroid function tests, serum free triiodothyronine (FT3), free thyroxine (FT4), and thyrotropin/thyroid-stimulating hormone (TSH) levels were determined by electrochemiluminescence immunoassay (ECLIA) using ECLusys FT3, FT4, and TSH (Roche Diagnostics, Basel, Switzerland). Serum antithyroglobulin antibody (TgAb) and antithyroid peroxidase antibody (TPOAb) levels were determined by ECLIA using an ECLusys Anti-Tg and Anti-TPO kit (Roche Diagnostics). Serum TRAb levels were determined by ECLIA using an ECLusys TRAb electrochemiluminescence immunoassay kit (Roche Diagnostics) [18]. Serum TSAb levels were measured using a TSAb radioimmunoassay and bioassay kit (Yamasa, Choshi, Japan). Serum TSAb levels were calculated as per the method described by Kamijo et al. [19], expressing the sample (S) in percentage corrected by the normal control (N, TSAb, 100%) and the positive control (P, TSAb, 750%): TSAb (%) = 100 + (S - N)/(P - N) × 650.

Thyroid function tests revealed a decreased serum TSH level of < 0.01 μIU/mL (reference range: 0.5–5.0 μIU/mL) and increased serum FT3, FT4, TgAb, and TPOAb levels of 5.19 pg/mL (reference range: 2.15–4.24 pg/mL), 2.43 ng/dL (reference range: 0.9–1.7 ng/dL), 37.0 IU/mL (reference range: < 27.0 IU/mL), and 252.0 IU/mL (reference range: < 15.0 IU/mL), respectively. Serum TRAb level was normal at < 0.8 IU/L (reference range: < 1.9 IU/L), although serum TSAb level was slightly increased at 122.0% (reference range: < 120.0%) (Table 1).

**Table 1:** Biochemical and thyroid function test data of patients who developed thyroid dysfunction after SARS-CoV-2 vaccination

		Days after the 1st SARS-CoV-2 vaccination^a^							
Patient 1	Reference range	Day 10	Day 16	Day 43	Day 92	Day 120	Day 148	Day 176	
CRP, mg/dL	< 0.14	NA	7.19	< 0.1	< 0.1	< 0.1	< 0.1	< 0.1	
TSH, μIU/mL	0.39–3.98	NA	< 0.01	12.84	3.69	3.78	3.68	3.57	
FT3, pg/mL	2.15–4.24	NA	5.19	1.44	2.59	3.05	2.94	2.91	
FT4, ng/mL	0.9–1.7	NA	2.43	0.59	0.92	0.95	0.99	1.10	
TRAb, IU/L	< 1.9	NA	< 0.8	NA	1.0	1.0	< 0.8	0.8	
TSAb, %	< 120.0	NA	122.0	NA	129.0	129.0	111.0	108.0	
TgAb, IU/mL	< 27.0	NA	37.0	NA	NA	14.0	14.0	13.0	
TPOAb, IU/mL	< 15.0	NA	252.0	NA	NA	72.0	35.0	12.0	
Symptoms		Fever (38.0 °C) Cervical pain Palpitations Fatigue		Resolution of all symptoms					
		Days after the 2nd SARS-CoV-2 vaccination^b^							
Patient 2	Reference range	Day 3	Day 28	Day 30	Day 51	Day 79	Day 87	Day 108	Day 138
CRP, mg/dL	< 0.14	NA	NA	4.48	0.22	0.12	0.14	0.12	< 0.1
TSH, μIU/mL	0.39–3.98	NA	< 0.01	< 0.01	< 0.01	0.56	< 0.01	< 0.01	1.65
FT3, pg/mL	2.15–4.24	NA	17.90	12.88	3.67	3.54	5.92	4.65	3.91
FT4, ng/mL	1.0–1.7	NA	5.13	4.18	1.54	1.32	1.67	1.64	1.58
TRAb, IU/L	< 1.9	NA	26.6	34.5	NA	34.5	NA	64.5	63.6
TSAb, %	< 120.0	NA	NA	1506.0	NA	269.0	NA	159.0	130.0
TgAb, IU/mL	< 27.0	NA	NA	38.0	NA	30.0	NA	96.0	62.0
TPOAb, IU/mL	< 15.0	NA	NA	77.0	NA	137.0	NA	220.0	174.0
Symptoms		Fever (37.8 °C) Pain in the left lobe of the thyroid gland	Fever (38.9 °C) Headache Pain in the left lobe of the thyroid gland Palpitations	Fever (37.5 °C) Pain in the right lobe of the thyroid gland Palpitations		Resolution of all symptoms	Fever (37.3 °C) Palpitations		Resolution of all symptoms
Imaging studies				TUS			TS		
Medications			MMI 15 mg/day	Discontinuation of MMI administration PSL 20 mg/day		Discontinuation of PSL	MMI 10 mg/day		MMI 5 mg/day

^a^Inoculated vaccine (BNT162b2, Pfizer, New York, NY, USA)

^b^Inoculated vaccine (mRNA-1273; Moderna, Cambridge, MA, USA)

*SARS-CoV-2* severe acute respiratory syndrome coronavirus 2, *CRP* C-reactive protein, *NA* not applicable, *TSH* thyroid-stimulating hormone, *FT3* free triiodothyronine, *FT4* free thyroxine, *TRAb* antithyroid-stimulating hormone receptor antibody, *TSAb* antithyroid-stimulating antibody, *TgAb* antithyroglobulin antibody, *TPOAb* antithyroid peroxidase antibody, *TUS* thyroid ultrasonography, *TS* thyroid scintigraphy with ^99m^technetium pertechnetate, *MMI* thiamazole, *PSL* prednisolone

**Fig. 1:**
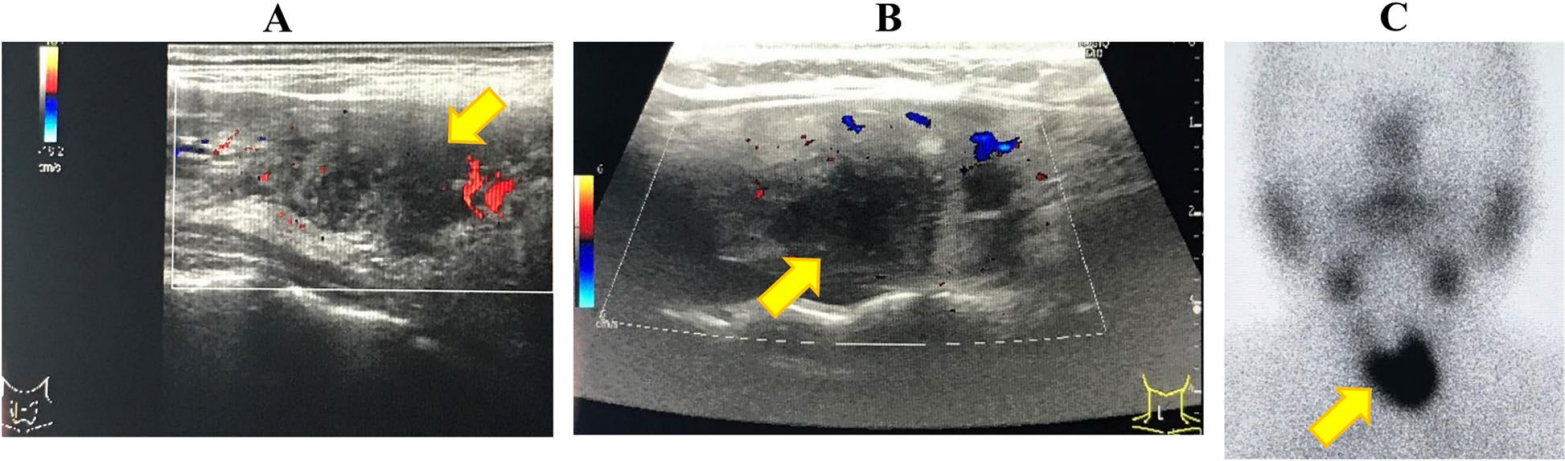
Thyroid ultrasonograms of two patients. Thyroid ultrasonograms of patient 1 (**A**) and patient 2 (**B**), indicating a focal hypoechoic area with decreased blood flow in the right lobe of the thyroid gland that coincided with the site of tenderness (arrow). Thyroid scintigram with ^99m^technetium pertechnetate of patient 2 (**C**) that was obtained at the recurrence of thyrotoxicosis shows the accumulation of an artificial radioactive element in the thyroid gland (arrow)

Physical examination revealed a slightly enlarged right lobe of the thyroid gland, which was tender upon palpation. Thyroid ultrasonography revealed a slightly swollen right lobe of the thyroid gland and a focal hypoechoic area with decreased blood flow (Fig. 1A), which coincided with the site of tenderness. The volume of the thyroid lobes was calculated using the volumetric formula reported by Suzuki et al. [20]. The width (cm), thickness (cm), and height (cm) of each lobe were measured during ultrasonography, and the volume (mL) of each lobe was calculated using the volume formula for ellipsoid (π/6 × width × thickness × height). The volumes of the right and left lobes were 8.8 mL and 5.9 mL, respectively. The patient was diagnosed with SAT and treated with oral acetaminophen (1000 mg/day for the first 5 days). Consequently, tenderness of the thyroid gland was alleviated although did not resolve. Fever (37.5 °C) recurred and fatigue occurred within 5–6 hours of oral acetaminophen administration. On day 6 of acetaminophen treatment, oral prednisolone (PSL) administration was initiated at a dose of 20 mg/day, which resolved the fever and tenderness of the thyroid gland. Subsequently, the PSL dose was tapered. On day 43 after SARS-CoV-2 vaccination, serum FT3 and FT4 levels decreased and TSH level increased (Table 1). The next day, i.e., on day 44 after SARS-CoV-2 vaccination, PSL was discontinued. On day 92 after vaccination, serum FT3, FT4, and TSH levels were within the normal range, even in the absence of thyroid hormone (TH) replacement therapy. After the discontinuation of PSL treatment, neither the symptoms nor the abnormal blood chemistry values recurred. On day 176 after vaccination, serum TSAb, TgAb, and TPOAb levels decreased to 108.0%, 13.0 IU/mL, and 12.0 IU/mL, respectively.

### Patient 2

A 36-year-old Japanese woman was healthy until SARS-CoV-2 vaccination (mRNA-1273; Moderna, Cambridge, MA, USA). She had no treatment or family history for thyroid disorders. The patient had not previously experienced adverse reactions to a vaccine or any drug. She had no smoking history. The patient received two vaccinations (mRNA-1273; Moderna), with a 28-day interval. The patient developed fever (38.9 °C) on the day following the second vaccination, which resolved the next day. However, on day 3 after the second vaccination, fever (37.8 °C) relapsed and cervical pain occurred. Subsequently, headache and palpitations occurred, cervical pain intensified, and fever (37.8 °C) persisted. The patient took aspirin purchased from a drug store. However, cervical pain, fever, and headache did not completely resolve, although they were temporarily alleviated. On day 28 after the second vaccination, the patient visited a nearby clinic where physical examination indicated fever (38.9 °C), a diffusely enlarged thyroid gland, and tenderness of the left lobe of the thyroid gland. The PCR test for COVID-19 was negative. The patient was diagnosed with GD based on the following thyroid function test results: serum TSH level, < 0.01 μIU/mL; serum FT3 level, 17.9 pg/mL; serum FT4 level, 5.13 ng/dL; and serum TRAb level, 26.6 IU/L. Although the clinic physician initiated the oral administration of thiamazole at 15 mg/day, left lobe thyroid gland pain, fever (≥ 38 °C), and palpitations did not improve. On day 2 after initiating the oral administration of thiamazole, i.e., on day 30 after the second vaccination, the patient visited Saitama Medical University Hospital for a consultation, where blood chemistry tests indicated a normal plasma WBC count of 7490 cells/μL and an elevated serum CRP level of 4.48 mg/dL. Thyroid function tests revealed a decreased serum TSH level of < 0.01 μIU/mL and increased serum FT3, FT4, TRAb, TSAb, TgAb, and TPOAb levels of 12.88 pg/mL, 4.18 ng/dL, 34.5 IU/L, 1506.0%, 38.0 IU/mL, and 77.0 IU/mL, respectively (Table 1).

Initially, the site of tenderness coincided with the left lobe of the thyroid gland. However, the site of tenderness changed during follow-up. At the time the patient sought medical attention at our hospital, tenderness occurred in the right lobe of the thyroid gland. Thyroid ultrasonography revealed a slightly enlarged right lobe of the thyroid gland and focal hypoechoic area with decreased blood flow (Fig. 1B), which coincided with the site of tenderness. Of special note were the facts that thyroid ultrasonography revealed reduced blood flow (an imaging characteristic of SAT) but not any findings suggestive of GD. The volume of both lobes was calculated using the aforementioned method [20]. The volumes of the right and left lobes were 10.2 mL and 8.4 mL, respectively. The patient was diagnosed with SAT because of severe tenderness at the thyroid gland site, elevated CRP levels, findings indicating thyrotoxicosis in thyroid function tests, and SAT-related findings during ultrasonography. On the day when the patient sought medical attention at our hospital, oral thiamazole administration (15 mg/day) was discontinued and oral PSL administration at a dose of 20 mg/day was initiated. We dithered over discontinuing thiamazole because she had increased TRAb levels, although we considered the presence of SAT based on ultrasonography findings and did not consider GD when attempting to make a precise diagnosis. We wanted to observe if improvements occurred by treating her only for SAT. After explaining this to the patient and obtaining her consent, we decided to discontinue thiamazole. Subsequently, the patient’s symptoms improved. Blood chemistry tests on day 51 after the second vaccination indicated normalized serum FT3 and FT4 levels (Table 1). The PSL dose was tapered when the symptoms resolved, and oral PSL administration was discontinued during week 7 after its initiation. However, palpitations and mild fever (37.0–37.3 °C) occurred 2 days after PSL discontinuation. Thyroid gland tenderness did not recur at that time or thereafter. Thyroid function tests at week 1 after oral PSL discontinuation revealed a decreased serum TSH level of < 0.01 μIU/mL, normal serum FT4 level of 1.67 ng/dL, and an increased serum FT3 level of 5.92 pg/mL. Thyroid scintigraphy with ^99m^technetium (Tc) pertechnetate, which was conducted on the same day, indicated the accumulation of the artificial radioactive element in the thyroid gland with an uptake rate of 2.21% 20 minutes after administration (Fig. 1C). At our hospital where this test is performed under conditions of a low TSH value and thyrotoxicosis, we diagnose destructive thyroiditis (SAT or silent thyroiditis) when the uptake rate is ≤ 0.5%, and GD when the uptake rate is ≥ 1%. Based on these findings, we diagnosed the patient with GD and prescribed 10 mg of oral thiamazole daily. Subsequently, the patient became euthyroid.

### HLA typing

We conducted HLA typing of two patients. DNA was extracted from the peripheral blood before outsourcing HLA typing to a clinical laboratory (Wakunaga Pharmaceutical Co., Ltd., Hiroshima, Japan). *HLA-A*, *-B*, *-C*, *-DQB1*, *-DPB1*, and *-DRB1* were genotyped using PCR based on the sequence-specific oligonucleotide method (Wakunaga Pharmaceutical Co., Ltd.). Written informed consent was obtained from both patients. The Institutional Review Board at Saitama Medical University Hospital approved the protocol for genetic analyses (approval number 2021-114, dated January 17, 2022). Table 2 shows the results of HLA typing that revealed the following HLA alleles: for patient 1, HLA-A*02:01/26:03, -B*35:01/40:01, -C*03:03/04:01, -DRB1*08:02/09:01, -DQB1*03:02/03:03, and -DPB1*02:01/05:01; and for patient 2, HLA-A*11:01/24:02, -B*15:01/35:01, -C*03:03/04:01, -DRB1*04:06/11:01, -DQB1*03:01/03:02, and -DPB1*02:01/05:01.

**Table 2:** HLA typing of two patients

	HLA alleles					
	*HLA-A*	*HLA-A*	*HLA-B*	*HLA-B*	*HLA-C*	*HLA-C*
Patient 1 with SAT	A*02:01^*b*^	A*26:03	B*35:01^*a*^	B*40:01	C*03:03	C*04:01^*a*^
Patient 2 with SAT and GD	A*11:01	A*24:02	B*15:01	B*35:01^*a*^	C*03:03	C*04:01^*a*^
	HLA alleles					
	*HLA-DRB1*	*HLA-DRB1*	*HLA-DQB1*	*HLA-DQB1*	*HLA-DPB1*	*HLA-DPB1*
Patient 1 with SAT	DRB1*08:02	DRB1*09:01	DQB1*03:02	DQB1*03:03	DPB1*02:01	DPB1*05:01^*b*^
Patient 2 with SAT and GD	DRB1*04:06	DRB1*11:01^*b*^	DQB1*03:01^*b*^	DQB1*03:02	DPB1*02:01	DPB1*05:01^*b*^

^a^Alleles associated with SAT

^b^Alleles associated with GD

*HLA* human leukocyte antigen, *SAT* subacute thyroiditis, *GD* Graves’ disease

## Discussion and conclusions

To the best of our knowledge, this is the first report on the HLA typing results of two Japanese patients who developed SAT and both SAT and GD after SARS-CoV-2 vaccination.

We could not completely rule out the acute exacerbation of Hashimoto’s thyroiditis (HT) in patient 1. Serum TPOAb and TgAb levels decreased to normal levels during follow-up. Nishihara et al. reported that serum TPOAb and TgAb levels may increase in SAT and decrease during follow-up [21]. Patient 1 exhibited a similar clinical course and was highly likely to have developed SAT. HT, when exacerbated acutely, causes thyrotoxicosis that results in persisting hypothyroidism [22]. However, patient 1 developed transient hypothyroidism with oral PSL administration and later became euthyroid, a condition that persisted thereafter. Therefore, we considered that patient 1 did not show the acute exacerbation of HT and was affected by SAT. The serum TSAb level increased slightly during the initial stage and then later decreased to normal levels during follow-up. Elevated serum levels of antithyroid antibodies in SAT patients are related mostly to antigen exposure during acute thyroid destruction [3, 23]. We presumed that this transient increase in serum TSAb level along with increased serum TPOAb and TgAb levels were laboratory changes that occurred with the onset of SAT.

Patient 2, who sought for medical attention at our hospital for the treatment of fever (37.5–38.9 °C) and pain in the thyroid gland, was diagnosed with SAT and was treated with oral PSL alone, resulting in improved serum TH levels and thyroid gland pain resolution. Therefore, we concluded that the patient developed SAT after SARS-CoV-2 vaccination, which increased serum TRAb levels. However, serum TH levels again increased thereafter. Patient 2 was diagnosed with GD by thyroid scintigraphy with ^99m^Tc pertechnetate during the follow-up of SAT. Notably, patient 2 had no treatment history for GD, had never shown GD symptoms (e.g., weight loss, finger tremors, and palpitations) and did not present with Graves’ orbitopathy before SARS-CoV-2 vaccination. However, patient 2 was not examined for serum TRAb and TH levels prior to SARS-CoV-2 vaccination, which impeded us to completely deny the possibility of GD development prior to vaccination. Moreover, we cannot rule out the likelihood that GD exacerbation and SARS-CoV-2 vaccination occurred in a temporal coincidence manner.

Since 2020, a number of case reports are available that reported on the onset of SAT and GD after COVID-19, both as single pathological entities. The results from a systematic review indicated that 13–64% of COVID-19 patients displayed thyroid dysfunction [24]. Additionally, COVID-19 but also SARS-CoV-2 vaccination is now considered to be involved in the onset of SAT and GD [10, 15]. Nevertheless, no study report is available that reported on the HLA typing outcomes and the concurrent onset of SAT and GD. SARS-CoV-2 vaccines can play a role in the pathogenesis of autoimmune diseases through various mechanisms (e.g., molecular mimicry, epitope spreading, polyclonal activation, and bystander activation) [25]. When the antigenic content of a vaccine shares structural similarities with autoantigens, immune responses to vaccine antigens could extend to host cells that exhibit similar self-antigens. On the other hand, recent studies investigating the incidence of SAT during COVID-19 found no changes in its incidence [26, 27]. These studies drive us to conjecture that COVID-19 and SARS-CoV-2 vaccines, when acting alone, do not trigger autoimmune diseases. However, molecular mimicry between thyroid proteins and infectious agents/vaccine antigens might trigger autoimmune responses in genetically susceptible individuals with specific HLA alleles (e.g., HLA-B*35 and HLA-C*04) [15, 28]. Additionally, not only specific HLA alleles but also other factors (e.g., tissue injury, prolonged inflammatory reaction) may be required to cause autoimmune disease [15, 26].

The HLA-B*35, -B*18:01, -DRB1*01, and -C*04:01 alleles are known to be involved in the pathogenesis of SAT [3]. The HLA-B*35 allele is that is known to augment susceptibility to SAT in both the Japanese and Caucasian populations, and approximately 70% of patients with SAT reportedly harbor the HLA-B*35 allele [4]. Both of our patients had the HLA-B*35:01 and -C*04:01 alleles which are strongly inferred to be involved in the postvaccination pathogenesis of SAT. Recently, Stasiak et al. reported that four patients who had developed SAT after COVID-19 [9] had HLA-B*35 alleles (two had HLA-B*35:01, one had HLA-B*35:03, and one had HLA-C*04:01). In addition, the HLA-B*35 and HLA-C*04:01 alleles were involved in the postvaccination pathogenesis of SAT in Caucasians [11, 12]. We also verified the co-presence of the HLA-B*35 and HLA-C*04:01 alleles in 2 Japanese patients, thus strongly suggesting the involvement thereof in the postvaccination pathogenesis of SAT.

Dong et al. reported that the HLA-DPB1*05:01 and *HLA-A2* HLA alleles are involved in the pathogenesis of GD in the Japanese population; harboring these two alleles is associated with increased risk for developing GD [6]. In our patients, patient 1 developed only SAT, whereas patient 2 developed both SAT and GD. Both patients had the HLA-DPB1*05:01 allele. Patient 1 had the HLA-A*02:01 allele although patient 2 did not have the *HLA-A2* allele. Dong et al. reported that possessing both HLA-DPB1*05:01 and *HLA-A2* alleles increases the risk for developing GD [6]. Notably, patient 1 who had both HLA alleles (HLA-DPB1*05:01 and *HLA-A2*) did not develop GD, although patient 2 who had only the HLA-DPB1*05:01 allele developed GD. We presume that GD in patient 2 was triggered after SARS-CoV-2 vaccination due to the involvement of other HLA alleles that would have intensified the GD susceptibility. Besides the HLA-DPB1*05:01 and *HLA-A2* alleles, *HLA-B46* and *HLA-Cw11* are reportedly involved in the pathogenesis of GD in the Japanese population [6]. Additionally, the HLA-DRB1*04:05 and HLA-DQB1*04:01 alleles reportedly confer susceptibility to GD in children [29]. However, patient 2, who developed both SAT and GD, had none of the aforementioned HLA alleles. The HLA-DRB1*03:01, HLA-DQB1*02:01, HLA-B*08:01, -B*39:06, -B*37:01, -C*07:01, -C*14:02, -C*03:02, -C*17:01, -DRB1*11:01, -DRB1*13:03, -DRB1*01:03, -DRB1*14:01, and -DQB1*03:01 alleles are associated with GD in Caucasians [30–33]. Patient 2—a Japanese female—had the HLA-DRB1*11:01 and HLA-DQB1*03:01 alleles among these alleles, suggesting the involvement thereof in the postvaccination pathogenesis of GD. The former allele has been reported in Caucasians [30] but has never been reported in Asians, while the latter has been reported in Asians including the Chinese, Koreans, and Thais [34, 35]. Nonetheless, both of the HLA-DRB1*11:01 and HLA-DQB1*03:01 alleles have never been reported to be associated with the postvaccination pathogenesis of GD not only in the Japanese but also Asian populations. At present, no evidence is available about 1) whether the HLA-DRB1*11:01 and HLA-DQB1*03:01 alleles are involved only in the postvaccination pathogenesis of GD, 2) whether these alleles are associated with the concurrence of SAT and GD, or 3) whether the combination thereof increases the risk of developing GD in the Japanese population. We intend to elucidate these issues in a larger scale clinical study.

Besides the HLA alleles, factors such as cluster of differentiation 40, cytotoxic T-lymphocyte-associated antigen 4, protein tyrosine phosphatase non-receptor type 22, Fc receptor-like protein 3, thyroglobulin, TSH receptor, zinc finger and AT-hook domain containing factor, forkhead box P3, interleukin (IL)-2 receptor alpha, IL-23 receptor, and interferon induced with helicase C domain 1 are known to be involved in the pathogenesis of GD [36–38]. We could not investigate whether our patients had one or more of these factors. Additionally, noncoding single nucleotide polymorphisms (SNPs) in intron 1 of the thyroid-stimulating hormone receptor (*TSHR*) gene are associated with GD [39]. Hiratani et al. [40] found that the SNP JST022302 and several adjacent SNPs in intron 7 of the *TSHR* gene were significantly associated with GD in the Japanese population and identified 3 haplotype blocks around intron 7 by linkage disequilibrium analysis. A single SNP haplotype (AATG [CT]6[TT]AG) in the haplotype block including JST022302 showed a significant association with GD in the haplotype case-control analysis. COVID-19 reportedly causes the upregulation of TSHR, which acts as an autoantigen in GD [41]. Presuming that such upregulation also occurs after SARS-CoV-2 vaccination, the risk for developing GD may be increased by the upregulation of TSHR autoantigen in individuals who have the *TSHR* gene haplotype mentioned above [40]. Nevertheless, we did not investigate these risk factors for GD other than the HLA alleles and therefore cannot rule out their association with the postvaccination pathogenesis of GD.

Nongenetic risk factors (e.g., iodine levels, presence of infections, psychological stress, sex susceptibility, smoking habits, thyroid damage, vitamin D levels, selenium levels, and presence of immune-modulating agents) are also involved in the pathogenesis of GD [36]. To date, serum vitamin D and selenium concentrations have never been evaluated in our patients. However, any nongenetic risk factor(s) other than SARS-CoV-2 vaccination and female sex were not considered as risk factors for GD pathogenesis in patient 2. We also aim to investigate genetic and nongenetic risk factors that could not be investigated this time.

In conclusion, we encountered two patients who developed thyroid disease after SARS-CoV-2 vaccination; one developed SAT and another developed both SAT and GD. The HLA alleles HLA-B*35:01 and HLA-C*04:01 appeared to be involved in the pathogenesis of postvaccination SAT. Moreover, the HLA-DRB1*11:01 and HLA-DQB1*03:01 alleles were speculated to be involved in the postvaccination pathogenesis of GD. The further accumulation of genetic and nongenetic evidence is required to elucidate the pathogenesis of SAT and GD that develop after vaccination for SARS-CoV-2.

## Data Availability

The datasets used and/or analyzed during the current study are available from the corresponding author on reasonable request.

## Abbreviations

COVID-19: Coronavirus disease 2019
CRP: C-reactive protein
ECLIA: Electrochemiluminescence immunoassay
FT3: Free triiodothyronine
FT4: Free thyroxine
GD: Graves’ disease
HLA: Human leukocyte antigen
HT: Hashimoto’s thyroiditis
IL: Interleukin
PCR: Polymerase chain reaction
PSL: Prednisolone
SAT: Subacute thyroiditis
SARS-CoV-2: Severe acute respiratory syndrome coronavirus 2
SNP: Single nucleotide polymorphism
^99m^Tc pertechnetate: ^99m^technetium pertechnetate
TgAb: Antithyroglobulin antibody
TH: Thyroid hormone
TPOAb: Antithyroid peroxidase antibody
TRAb: Antithyroid-stimulating hormone receptor antibody
TSAb: Antithyroid-stimulating antibody
TSH: Thyroid-stimulating hormone/thyrotropin
TSHR: Thyroid-stimulating hormone receptor
WBC: White blood cell
